# A clinical tool to predict *Plasmodium vivax* recurrence in Malaysia

**DOI:** 10.1186/s12879-017-2868-9

**Published:** 2017-12-08

**Authors:** Norliza Mat Ariffin, Farida Islahudin, Endang Kumolosasi, Mohd Makmor-Bakry

**Affiliations:** Quality Use of Medicine, Faculty of Pharmacy, Universiti Kebangsaan Malaysia, 50300 Kuala Lumpur, Malaysia

**Keywords:** Malaria, Recurrence, *Plasmodium vivax*, Primaquine, Clinical tool

## Abstract

**Background:**

Recurrence rates of *Plasmodium vivax* infections differ across various geographic regions. Interestingly, South-East Asia and the Asia-Pacific region are documented to exhibit the most frequent recurrence incidences. Identifying patients at a higher risk for recurrences gives valuable information in strengthening the efforts to control *P. vivax* infections. The aim of the study was to develop a tool to identify *P. vivax*- infected patients that are at a higher risk of recurrence in Malaysia.

**Methods:**

Patient data was obtained retrospectively through the Ministry of Health, Malaysia, from 2011 to 2016. Patients with incomplete data were excluded. A total of 2044 clinical *P. vivax* malaria cases treated with primaquine were included. Data collected were patient, disease, and treatment characteristics. Two-thirds of the cases (*n* = 1362) were used to develop a clinical risk score, while the remaining third (*n* = 682) was used for validation.

**Results:**

Using multivariate analysis, age (*p* = 0.03), gametocyte sexual count (*p* = 0.04), indigenous transmission (*p* = 0.04), type of treatment (*p* = 0.12), and incomplete primaquine treatment (*p* = 0.14) were found to be predictors of recurrence after controlling for other confounding factors; these predictors were then used in developing the final model. The beta-coefficient values were used to develop a clinical scoring tool to predict possible recurrence. The total scores ranged between 0 and 8. A higher score indicated a higher risk for recurrence (odds ratio [OR]: 1.971; 95% confidence interval [CI]: 1.562–2.487; *p* ≤ 0.001). The area under the receiver operating characteristic (ROC) curve of the developed (*n* = 1362) and validated model (*n* = 682) was of good accuracy (ROC: 0.728, 95% CI: 0.670–0.785, *p* value < 0.001, and ROC: 0.766, 95% CI: 0.700–0.833, *p*-value < 0.001, respectively). In both the developed and validated models, area under the ROC curves showed no significant difference in predicting recurrence based on the constructed scoring mechanism (*p* = 0.399; Z-value: −0.8441; standard error: 0.045).

**Conclusions:**

The developed model to predict recurrence was found to be of good accuracy and could be a useful tool in targeting patients at a higher risk for recurrence for closer monitoring during follow-up, after treatment with primaquine.

## Background

Malaria caused by *Plasmodium vivax* affects an estimated 16 million cases worldwide [1]. In 2015, *P. vivax* infection was responsible for approximately 3100 deaths globally, with 86% occurring outside Africa, mainly in South-East Asia and the Eastern Mediterranean region [1]. Asymptomatic parasite carriage and early gametocyte production have contributed to the vast cases of malaria by *P. vivax* infection [2]. Recurrence either through reinfection, relapse and recrudescence occurs frequently [1, 2]. Furthermore, the ability of the parasite to relapse in the weeks and months following a primary parasitaemia via the dormant liver-stage known as hypnozoites [3], also contributes to a major challenge in eradicating the infection. These characteristics pose a considerable burden in managing *P. vivax* infections.

At present, primaquine plays a unique role in the prevention and treatment of *P. vivax* malaria, as it is the only FDA-licensed drug capable of clearing the intra-hepatic schizonts and hypnozoites of *P. vivax* [4]. Further, the use of primaquine is recommended in combination with artemisinin or chloroquine. However, the clinical effectiveness of primaquine is limited by a long treatment-course of 2 weeks and potential haemolytic adverse events. The current dosage guideline for primaquine is 30 mg/day, for 2 weeks, in patients not exhibiting glucose-6-phosphate (G6PD) deficiency. An intermittent primaquine regimen of 0.75 mg base/kg body-weight, once a week, for 8 weeks, is given to those who are found to have G6PD deficiency. Primaquine is contraindicated during pregnancy, since the G6PD status of the foetus would be unknown; pregnant patients are given a full dose after delivery taking into account their G6PD status [2, 5, 6]. Owing to the uniqueness of primaquine in eliminating recurrences in *P. vivax* infection, ensuring the effectiveness of the drug in the long run is vital; this is especially an area of concern in regions where malaria caused by *P. vivax* occurs frequently, in order to reduce recurrence of infection.

Recurrence of malaria due to *P. vivax* differs across various geographic regions [7]. Interestingly, South-East Asia and the Asia-Pacific region are documented to exhibit the most frequent recurrence in the form of relapse incidence-rates [7]. In Malaysia, a 1-year follow-up is necessary to identify recurrence following primary infection and completion of primaquine therapy [8]. Although shorter recurrence periodicity has been noted in certain parts of the world, including Malaysia [6–8], a 1-year follow-up is recommended to reduce risk of local transmission within the area [8]. However, of the total 5955 patients with *P. vivax* infection registered in the national malaria case registry, between 2011 and 2015, only 2037 cases (34.2%) were successfully followed-up to 6 months after completing the primaquine treatment [8]. The remaining cases were unsuccessfully monitored due loss of patient contact, lack of information on the primaquine treatment, or other unclear data. This lack of a thorough follow-up post completion of treatment could impair the national effort to eradicate malarial recurrence occurring in *P. vivax* infections.

Various factors are known to affect recurrence [9]. The incidence rate of 836 relapse per 100,000 person-days observed in Malaysia, was predicted based on factors such as climate, mode of malaria transmission, and vector conditions [6]. Other factors such as male-gender, shorter symptom-periods, and higher parasitaemia in initial infections have also resulted in a higher risk for recurrence [10]. The efficacy of primaquine in the prevention of *P. vivax* recurrence varies based on geographic regions [11, 12], with tropical strains demonstrating a higher ability of recurrence [9]. Inadequate dosing [13], as well as non-adherence to the full recommended course of primaquine therapy [14] are also risk factors. Identifying the risk for recurrence gives valuable information in strengthening the efforts to control the *P. vivax* malaria transmission. In view of the difficulty in managing recurrences in *P. vivax*-infected patients, a robust way to predict and overcome recurrence is required. Due to the year-long monitoring of recurrence required in patients infected with *P. vivax*, a tool to ascertain high-risk patients in the clinical setting would be valuable. Identifying recurrence-predictors based on national data could potentially be used to identify patients who are at a greater risk for recurrence. This could provide a basis for ensuring stricter monitoring of primaquine treatment in susceptible patients. Therefore, in this study, we aimed to develop a tool to identify *P. vivax*-infected patients who are at a higher risk of recurrence in Malaysia.

## Methods

### Study design

A retrospective study was performed from 2011 to 2016. Data were obtained through the National Malaria Case Registry (NMCR) under the Vector Borne Disease Sector, Disease Control Division, Ministry of Health, Malaysia. Registration of malaria cases is compulsory in Malaysia and is available as a centralised electronic database. Both private and public institutions are required to inform vector control units of malaria cases. The cases are assessed for appropriate information, and entered into the registry by the local vector control units. From the registry, patients diagnosed with *P. vivax* infection and treated with primaquine were included in this study. Patients with missing data and those who were not successfully monitored for at least 6 months after discharge were excluded from the study.

In order to develop a prediction model for the risk of recurrence, the study sample was divided into 2 groups. Two-thirds of the sample-size was used to develop the prediction model. The remaining one-third was used for validation [15, 16]. The study participants were randomized into the 2 sample-groups in order to minimize bias [16]. Randomization was performed using the IBM SPSS Statistics software (version 23; IBM Corp., Armonk, NY).

### Ethical considerations

The study was registered under the National Medical Research Registration and ethical approval was obtained from the National Medical Research Ethics Committee [ID: NMRR-17-562-34,981). The current work was also approved by the Vector Borne Disease Sector, Disease Control Division, Ministry of Health, Malaysia.

### Data collection

Characteristics of variables captured from the National Malaria Case Registry database was grouped into 3 main categories; patient characteristics, disease characteristics, and treatment characteristics. Data collected for patient characteristics were age, gender, nationality, WHO region countries, pregnancy and G6PD status, case locality, and status of case-locality. G6PD was determined using a blood test as part of standard hospital management. Disease characteristics included length of stay during the initial hospital admission, survival during the initial hospital admission, previous malaria infection, presence of gametocyte during the first day of admission, sexual and asexual parasite counts on the first day of admission, the week of infection-onset, severity, and type of transmission of malaria. Week of infection-onset is based on the 52-week calendar. Severity was defined based on the WHO categories of non-severe and severe [11, 12]. Treatment characteristics included the type of primaquine combination therapy (chloroquine or artemisinin-based combination therapies), and completion of primaquine treatment. The dosage of primaquine was 30 mg/day, for 2 weeks, in patients without G6PD deficiency. An intermittent primaquine regimen of 0.75 mg base/kg body-weight, once a week, for 8 weeks, was given to those who were found to have G6PD deficiency. Pregnant patients are given a full dose upon delivery taking into account their G6PD status. The treatment completion rate was assessed through a primaquine adherence card, which was given to the patient on discharge as part of standard treatment. All the defined characteristics were considered as possible predictors of recurrence.

### Definitions used in the study

Patients were categorised based on their nationalities into defined 6 WHO regions: African Region, Region of the Americas, South-East Asia Region, European Region, Eastern Mediterranean Region, and Western Pacific Region [17]. According to the Vector Borne Disease Sector, Ministry of Health, Malaysia, ‘case locality’ refers to the source of malaria infection: rural, urban, agricultural sites, and logging or federal/state land. The ‘status of case-locality’ refers to the defined magnitude of infection within the area, as set by the Vector Borne Disease Sector, Ministry of Health, Malaysia: prone, problematic, or free of malaria infection. ‘Previous infection’ was noted based on a detailed patient medical history and patient interviews by health inspectors during admission. Type of transmission was divided into 4 categories, which refer to the main cause of infection: imported, indigenous, introduced, and relapse, based on the WHO definitions [18]. ‘Indigenous transmission’ is defined as locally acquired malaria infection, in an area where malaria is prevalent. It is also defined as malaria contracted locally with no evidence of importation and no direct link to transmission from an imported case [18]. Infections which are acquired outside a local area (in this case Malaysia) are termed as ‘imported transmission’. ‘Introduced transmission’ refers to malaria contracted locally, with strong epidemiological evidence linking it directly to a known imported case (first-generation local transmission) [18]. ‘Relapse transmission’ is defined as recurrence of parasitaemia in *P. vivax* or *P. ovale* following primary infection and occurs when blood-stage infection has been eliminated but hypnozoites persist in the liver [18]. All cases categorised under relapse will be termed recurrence due to the inability to differentiate between relapse, reinfection, or recrudescence.

Clinical outcome was measured by recurrence during a 6- to 12-month follow-up, as determined from a positive blood film malaria parasite (BFMP) test. Patients were actively monitored for presence of parasites on a monthly basis by the health district office as recommended by the current clinical practice in the management of *P. vivax* [19]. A positive recurrence was defined as a presence of parasite during the follow-up.

### Statistical analyses

Statistical analysis was performed using the IBM SPSS Statistics software (version 23; IBM Corp., Armonk, NY). Two-third of the sample was used for developing the scores for risk of recurrence [15, 20]. Univariate and multivariate logistic regression were used to determine whether the predictors (patient, disease, and treatment characteristics) affected recurrence. In the univariate analysis, the variables, patients were divided based on the country-of-origin as Malaysians and non-Malaysians, while the WHO regions were dichotomised into Western-Pacific and non-Western-Pacific regions. Independent predictors were evaluated by univariate analysis at a level of significance of *p* ≤ 0.1 [20]. These predictors were then evaluated by multivariate regression, where continuous data (age and gametocyte counts) were stratified and analysed as categorical data based on the median values [15, 20].

The presence of correlation between predictors or multi-collinearity was excluded in the final model to enhance the prediction of significant predictors [20, 21]. Variables with *p* values ≤0.15 were considered as independent indicators of the risk of recurrence and retained in the final model [22]. This value was also chosen to take into account variables that are known to be important in clinical management based on professional clinical judgment [22, 23]. This was performed through consultation with 3 clinical researchers. From the final logistic regression model, the value of beta-coefficients was used as a basis for score values [20, 22]. In order to calculate the scores for risk of recurrence, beta-coefficient values were divided by a reference value of 0.66 (the reference was obtained by identifying the lowest beta-coefficient value; in this study the beta coefficient value for age was the lowest, i.e. 0.66), and rounded to the nearest integer [15].

Predictor scores were then assigned to two-thirds of the sample. A logistic regression and the Hosmer-Lemeshow test were performed to determine the calibration of the model using risk scores that were developed [15, 20]. To distinguish the ability of the risk-prediction model to predict risk of recurrence using the total scores, the area under the receiver operating characteristic (ROC) curve was analysed [15, 20].

One-third of the remaining sample-size was then used for validation and optimisation of the final model [15, 20]. Validity of this risk prediction score model was performed by assigning the developed total risk score for each patient. The predictors were tested for their predictive capacity and accuracy using area under the ROC curve [16, 20]. The performance of predicting modelling using area under the ROC was in accordance with previous study [22, 23]. Accuracy of the prediction, based on the area under the ROC curve was classified as previously described: 0.7–1.0 as ‘good’, and <0.7 as ‘poor’ [22, 23]. Trade-off values from ROC curves were used to categorise the values into high- and low-risk of recurrence [16].

## Results

### Demographics

**Fig. 1:**
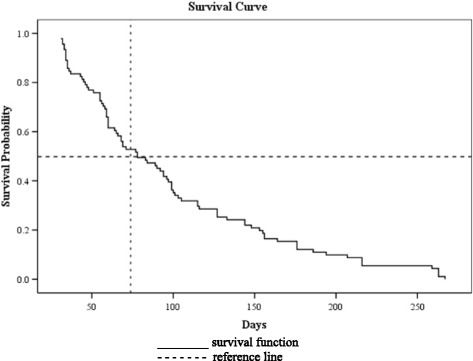
Kaplan Meier survival curve for recurrence of *P. vivax* malaria (2011–2016). Survival function is defined as the probability of recurrence not occurring

A total of 2044 patients infected with *P. vivax* between 2011 and 2016 were included for development and validation of total scores in predicting presence and absence of recurrence (Table 1). The number of days for recurrence of *P. vivax* infection ranged between 31 and 267 days after the start of antimalarial treatment. In a survival analysis, the overall median time to recurrence obtained by the Kaplan-Meier curve was 78 days (95% CI: 86.1–112.6), which means 50% of the recurrence cases occurred in less than 78 days after start of the treatment (Fig. 1).

**Table 1:** Characteristic of malaria patients in the study population (*n* = 2044) from 2011 to 2016

Patient characteristic	n	Percentage (%)
Age, years, (mean, 95% CI) (range)	28.0, 27.3–28.7	2 weeks −80 years
Gender (n) (%)		
Male	1558	76.2
Female	486	23.8
Nationality (n) (%)		
Malaysian	1310	64.1
Non-Malaysian	734	36.0
WHO Region (n) (%)		
Africa	1	0.1
South-East Asia	522	25.5
Western Pacific	1367	66.8
Eastern Mediterranean	152	7.4
Unknown	2	0.1
Glucose-6-phosphate-dehydrogenase (G6PD) (n) (%)		
Deficient	126	6.2
Normal	1252	61.2
Unknown	666	32.6
Pregnancy (n) (%), total = 486		
No	448	92.2
Yes	38	7.8
Case locality		
Urban	366	17.9
Rural	1090	53.5
Unknown	588	28.7
Status of case locality (n) (%)		
Free	829	40.6
Prone	164	8
Problematic	1033	50.5
Unknown	18	0.9
Disease characteristic		
Length of stay (days) (mean, 95% CI) (range)	6.1, 5.9–6.3	0–45
Previous malaria infection (n) (%)		
No	1418	69.4
Yes	454	22.2
Unknown	172	8.4
Presence of gametocyte during admission (n) (%)		
Positive	808	39.5
Negative	579	28.3
Unknown	657	32.1
Gametocyte/μL on admission (mean, 95% CI) (range)	857.4, 741–972	1–19,642
Asexual/μL on admission (mean, 95% CI) (range)	6402, 5458–7346	1–732,200
Week onset (week) (mean, 95% CI) (range)	20.53, 19–21	0–52
Severity (n) (%)		
Non-severe	1961	95.9
Severe	55	2.7
Unknown	28	1.4
Malaria transmission type (n) (%)		
Imported	739	36.2
Indigenous	1195	58.5
Introduced	47	2.3
Relapse before	63	3.1
Treatment characteristic		
Type of antimalarial drugs (n) (%)		
CQ plus PQ	1373	67.2
ACT plus PQ	669	32.7
No treatment	2	0.1
Status of PQ treatment (n) (%)		
Complete	2014	98.5
Incomplete	14	0.7
Unknown	16	0.8

*G6PD* Glucose-6-phosphate dehydrogenase, *CQ* Chloroquine, *PQ* Primaquine, *ACT* Artemisinin-based combination therapies

Among the study population, 553 (27.1%) patients were children aged <18 years, while 1491 (72.9%) were adults aged ≥18 years (Table 1). The majority of the patients were Malaysian (*n* = 1310, 64.1%), and the remaining were foreigners (*n* = 734, 36%): Indonesia (299, 14.6%), Pakistan (154, 7.5%), Myanmar (72, 3.5%), Nepal (64, 3.1%), India (53, 2.6%), Philippines (45, 2.2%), Bangladesh (31, 1.5%), Cambodia (7, 0.3%), Thailand (3, 0.1%), Vietnam (3, 0.1%), China (1, 0.1%), and Nigeria (1, 0.1%). Based on the WHO region classification, the participants were further stratified into those belonging to Africa (1, 0.1%), Europe (2, 0.1%) South-East Asia (522, 25.5%), Western Pacific (1367, 66.8%), and Eastern Mediterranean (152, 7.4%) [17]. G6PD was determined using a blood test and was performed for all patients; however, the G6PD status was noted as ‘unknown’ for 666 (32.6%) patients, where the results were not available by the time the patient was discharged from the hospital. No deaths were reported in the study.

Previous malaria infection was identified in 454 (22.2%) patients; however, the type of malaria identified was not noted. In a small number of patients (172, 8.4%), previous malaria infection was not confirmed due to the lack of medical information. Among the type of malaria transmission, 36.2% of the cases were imported (*n* = 739), 58.5% indigenous (*n* = 1195), 2.3% introduced (*n* = 47), and 3.1% relapse (*n* = 63).

During the study duration, it was noted that 2 patients (0.1%) were not administered pharmacological treatment due to refusal for further management of treatment during active case surveillance. The status of treatment completion was also unknown in a small percentage of patients (*n* = 16, 0.8%) as they could not be traced during the 6-month follow-up period based on their most recent addresses.

### Predictors of recurrence

Two-thirds (*n* = 1362) of the study participants were used for development of the predictor scores. A univariate logistic regression analysis showed that patient (age, gender, nationality, WHO region countries, pregnancy, case locality (rural), and status of case-locality (prone); disease (gametocyte count and malaria transmission type); and treatment characteristics (the antimalarial treatment used) were risks of recurrence (Table 2). A strong multi-collinearity was found among nationalities, Western-Pacific countries, pregnancy, and case locality; these were excluded from the final model. When all variables with a *p*-value of ≤0.1 in the univariate analysis were tested in a multivariate analysis, age, gametocyte sexual count, indigenous type of transmission, type of treatment used, and incomplete primaquine-treatment were found to be predictors of recurrence after controlling for other confounding factors; these were included in the final model (Table 3).

**Table 2:** Univariate logistic regression on risk of recurrence from 2011 to 2016 (*n* = 1362)

Patient characteristic (reference)	Recurrence (*n* = 127)	Beta	Odds Ratio	95% CI		*P*-value
Age (≥ 27 years)	83	–	1.00	–	–	–
< 27 years	44	0.62	1.90	1.14	3.17	0.00
Gender (Female)	52	–	1.00	–	–	–
Male	75	0.92	0.40	0.28	0.58	0.00
Malaysian (Non-Malaysians)	16	–	1.00	–	–	–
Malaysians	111	1.35	3.87	2.30	6.51	0.00
Region (Non-Western Pacific)	13	–	1.00	–	–	–
Western Pacific	114	1.37	3.95	2.28	6.82	0.00
G6PD (Normal)	125	–	1.00	–	–	–
Deficient	2	−1.18	0.31	0.07	1.27	0.12
Pregnant (Non-pregnant)	44	–	1.00	–	–	–
Pregnant	8	0.89	2.44	1.05	5.66	0.04
Case locality (Urban)	0	–	1.00	–	–	–
Rural	104	1.33	3.77	1.89	7.55	0.00
Agriculture	23	0.70	2.01	0.84	4.77	0.11
Status of case locality (Free)	32	–	1.00	–	–	–
Prone	0	−1.08	0.34	0.08	1.43	0.14
Problematic	95	1.03	2.73	1.78	1.43	0.00
Disease characteristic (reference)						
Length of stay (days) (range)	1–14 days	−0.01	0.99	0.95	1.04	0.68
Previous infection (no)	97	–	1.00	–	–	–
Yes	30	−0.27	0.76	0.48	1.21	0.25
Presence of gametocyte (negative)	37	–	1.00	–	–	–
Positive	90	0.24	1.27	0.84	1.92	0.27
Gametocyte (<80 parasite/μL)	44	–	1.00	–	–	–
≥ 80 parasite/μL	83	0.47	1.60	1.07	2.40	0.02
Asexual (<2700 parasite/μL)	60	–	1.00	–	–	–
≥ 2700 parasite/μL	67	0.068	1.07	0.74	1.54	0.71
Week onset (weeks)	1–52	0.00	1.00	0.98	1.01	0.49
Severity (Non-severe)	127	–	1.00	–	–	–
Severe infection	0	−18.52	0.00	0.00	.	1.00
Malaria transmission type						
(Non-indigenous)	10	–	1.00	–	–	–
Indigenous	107	1.21	3.35	2.06	5.50	0.00
Relapse before	10	1.50	4.50	1.82	11.08	0.11
Treatment characteristic (reference)						
Treatment type (ACT plus PQ)	106	–	1.00	–	–	–
CP plus PQ	21	0.99	2.70	1.66	4.39	0.00
Status of treatment (Complete)	125	–	1.00	–	–	–
Incomplete	2	1.445	4.242	0.90	20.02	0.068

*ACT* Artemisinin-based combination therapies, *WHO* World Health Organization, *G6PD* Glucose-6-phosphate dehydrogenase, *CP* Chloroquine, *PQ* Primaquine

**Table 3:** Multivariate logistic regression on risk of recurrence from 2011 to 2016 (*n* = 1362)

Characteristic (reference)	Beta	Odds Ratio	95% CI		*P*-value	Score
Age (≥ 27 years)						
Less than 27 years	0.66	1.93	1.07	3.51	0.03	1
Gametocyte (<80 parasite/μL)						
≥ 80 parasite/μL	1.53	4.60	1.06	19.84	0.04	2
Malaria transmission type (Non-indigenous)						
Indigenous	0.88	2.42	1.03	5.69	0.04	1
Treatment type (ACT plus PQ)						
CP plus PQ	0.96	2.61	0.78	8.74	0.12	2
Status of treatment (Complete)						
Incomplete	1.29	4.48	0.89	20.02	0.14	2
Total score						8

*ACT* Artemisinin-based combination therapies, *CP* Chloroquine, *PQ* Primaquine

**Fig. 2:**
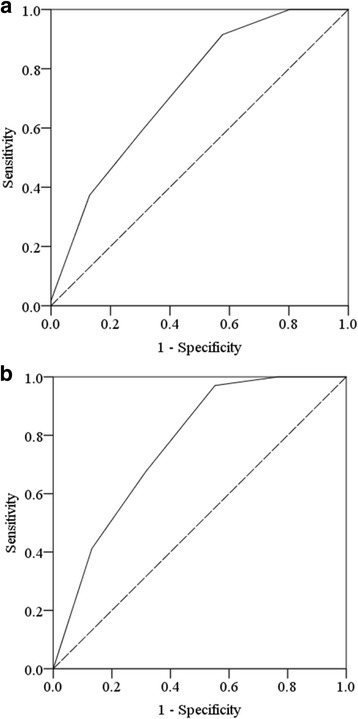
Area under the ROC curve. (**a**) development of the risk of recurrence prediction scores, AUC: 0.728. (**b**) validation of the risk of recurrence prediction scores, AUC: 0.766

Based on the beta-coefficient values (age, gametocyte counts, indigenous transmission type, the combination of chloroquine and primaquine treatment, and incomplete primaquine-treatment), the total scores ranged between 0 and 8. The score-values were assigned for each patient and a regression analysis demonstrated that an increase in 1 score unit increased the risk of recurrence by 1.971 times (95% CI for OR: 1.562–2.487; *p* ≤ 0.001). Therefore, an increase in the total risk score increased the likelihood of recurrence. The Hosmer-Lemeshow goodness-of-fit test showed that the model had a *p*-value of 0.221, indicating that the model did not misrepresent the data [22]. The performance and accuracy of the model in predicting both presence and absence of recurrence was measured by the area under the ROC curve of the developed model, which was 0.728 (95% CI: 0.670–0.785; *p* < 0.001; Fig. 2A), indicating that the accuracy of the model was good [23]. The trade-off value of the score was 3.5, which represents 91.5% sensitivity and 57.7% specificity in predicting the presence or absence of recurrence (Table 4). Score values were then categorised into high-risk (>3.5) and low-risk (≤3.5) of recurrence [16].

**Table 4:** Validation and optimization of the model in predicting *P. vivax* recurrence

Model	n	Trade-off value	Sensitivity (%)	Specificity (%)	AUC ROC	95% CI		*P*-value	*P*-value
Development	1362	3.5	91.5	57.7	0.728	0.670	0.785	0.00	0.399
Validation	682	3.5	97.1	55.2	0.766	0.700	0.833	0.00	

*AUC ROC* Area under the receiver operating characteristic curve

We then performed a validation and optimisation test of the model on one-third (*n* = 682) of the study participants. The score values were assigned for all patients; regression analysis demonstrated that an increase in 1 score unit increased the risk of recurrence by 2.208 times (95% CI for OR: 1.585–3.076; *p* ≤ 0.001). The area under the ROC curve demonstrated the model was reliable with a value of 0.766 (95% CI: 0.700–0.833; *p* < 0.001; Fig. 2B). The model gave a good recurrence prediction based on the developed prediction score model. The trade-off value score of 3.5 represented 97.1% sensitivity, with 55.2% specificity, in predicting presence or absence of recurrence (Table 4). Score values were then categorised into high-risk (>3.5) and low-risk (≤3.5) of recurrence [16]. In both the developed and validated models, area under the ROC curves showed no significant difference in predicting recurrence based on the constructed scoring mechanism (*p* = 0.399, Z-value: −0.8441, standard error: 0.045; Table 4), demonstrating that the tool was optimised.

Further evaluation of the constructed scores was performed by comparing it to the predictive ability of the logistic regression model. The ROC of the regression model was calculated by dividing the dataset into 2 parts: two-thirds and one-third, similar to that used in the constructed scores using beta coefficients. The performance and accuracy of the logistic regression model in predicting recurrence that was based on two-thirds of the sample had an area under the ROC curve of 0.715 (95% CI: 0.655–0.775; *p* < 0.001). When the logistic regression model was applied to one-third of the sample, the area under the ROC curve was 0.723 (95% CI: 0.642–0.804; p < 0.001). A comparison of the 2 ROC values of two-thirds of the sample using the logistic regression model and the constructed scores found that the difference between them was not significant (ROC 0.715 vs ROC 0.728 respectively; *p* = 0.81, Z-value: 0.243, standard error: 0.054). Similarly, the difference in the ROC values of one-third of the sample between the logistic regression model and constructed scores was also non-significant (ROC 0.723 vs ROC 0.766 respectively; *p* = 0.53, Z-value: 0.636, standard error: 0.068). Therefore, the ROC values for both the logistic regression model and constructed scores were found to be comparable. This demonstrates that the constructed scores were optimised.

## Discussion

A growing body of evidence shows that malaria caused by *P. vivax* is no longer thought of as a benign and rarely fatal disease [24, 25]. Despite various preventative measures, complete elimination of *P. vivax* has become most challenging due to its ability to recur in the weeks and months following a primary parasitaemia [24, 25]. In Malaysia, the incidence of *P. vivax* is still significant as evident from the number of infections observed in the current study. Further, due to urbanization and immigration of foreign workers, the number of infections among non-Malaysians residing in Malaysia is also high [8]. Although *P. vivax* infects patients of all ages, the present work demonstrates that it is prevalent mostly among adolescent males, similar to that reported previously [10]. The socio-economic impact of this, if left unmonitored, would significantly affect the country’s development [26]. Therefore, close monitoring of patients with *P. vivax* infection and identifying those at a higher risk of recurrence is vital in order to better control and eradicate the disease.

Identifying risk of recurrence of *P. vivax* infections is a challenging task. The current study successfully demonstrated that predictors of recurrence of *P. vivax* infection in the local settings were younger age, higher gametocyte on admission, indigenous transmission, combinatorial treatment with chloroquine and primaquine, as well as incomplete primaquine treatment. This is similar to a previous study which demonstrated that the risk factors for recurrence were geographic region, male-gender, and younger age [9]. Furthermore, as primaquine is currently the only licensed radical-treatment for hypnozoites [4], incomplete primaquine treatment also contributes to risk of recurrence in *P. vivax* infection [27, 28]. The present data suggests that the current predictor model developed from our local database provides a simple way to predict malaria recurrence in patients with *P. vivax* infections with good accuracy. Scores above 3.5 achieved in the current model suggests a higher possibility of incidence of recurrence. The use of clinical scores has recently gained popularity as a guide for health personnel to monitor patients at a higher risk for certain diseases [15, 16]. This is especially useful when large numbers of patients are present and longer monitoring periods are required [2], as in the case of *P. vivax* infection.

Among the risk factors, the present model predicts that demographic characteristics, such as age, affect recurrence. The model predicts that a higher recurrence rates occurs among younger patients, which was similarly demonstrated in another study [29]. The frequency of recurrence in individuals aged 12–49 years has been proportionally higher than in other age groups [10]. Thus, it is suggested that older adults acquire much more rapid and intense immunity against *P. vivax* [30], a finding that is consistent with local data. However, *P. vivax*-infected individuals above 70 years are also at a risk of higher mortality [30], suggesting age as a possible confounding factor.

A greater number of gametocyte counts among patients with indigenous infections signify a challenge in reducing recurrence. In areas where *P. vivax* predominantly affects local people, the consequence of producing delayed blood-stage infections within a community could be serious [27, 31]. This has proven to lead to asymptomatic infections, which may remain undetected and serve as a reservoir in transmitting the disease to mosquitoes [32–34], and subsequently to individuals.

There has been an alarming urgency for better treatment, spurred by the development of resistance to the older antimalarial drugs [33, 35]. The availability of primaquine as the sole drug, recommended to treat hypnozoites, is also a major concern. The use of chloroquine in combination with primaquine was observed to greatly affect *P. vivax* infection with incidence of recurrence occurring at a higher rate throughout the 1-year routine follow-up compared to the use of artemisinin combinations. A recent work conducted in Sabah, is indicative of the waning efficacy of chloroquine combination despite adequate blood concentrations of the drug [33, 36]. This has led to the introduction of co-artemether and primaquine combination for treatment of *P. vivax*. Apart from inefficacious primaquine treatment, inadequate knowledge on malaria treatment among aboriginal people, could lead to non-compliance to primaquine treatment following discharge. Incomplete primaquine treatment could lead to recurrence [28], especially when non-compliance exists in high-risk groups [33, 35]. Therefore, daily monitoring of primaquine administration to ensure primaquine treatment is completed is a better approach in a high-risk population. This could ensure sustainable efficacy of primaquine in management of *P. vivax* infections.

In view of the concern of recurrence with *P. vivax* infection, the ability to predict high-risk patients in our population will largely benefit the community. To the best of our knowledge, this is the first time a clinical recurrence prediction tool is developed using national data. Evidently, this tool was found to be a good predictor, and could be used upon discharge by healthcare personnel to identify high-risk of recurrence among treated patients. Patients lost to follow-up during the 1-year monitoring period is a limitation of the current system [19]. However, with the use of a clinical recurrence prediction tool, greater attention could be given to those that are at a higher risk of recurrence. We propose enforcing a strict malaria program in patients, with scores >3.5, who are at a higher risk of recurrence. This can be implemented through patient education of the complications associated with *P. vivax* recurrence and the importance of close-monitoring prior to discharge. Health personnel could also focus on efforts to ensure patients are re-educated during their monthly monitoring to improve patients’ understanding of *P. vivax* malaria, thereby reducing the loss to follow-up.

Despite successfully developing and validating the scoring tool, there were several limitations to the current study. It is possible that we have not identified all patients with a recurrence, due to loss to follow-up of a significant number of cases. This is especially a concern as some patients tend to demonstrate recurrence, months after the initial infection. Furthermore, a much more precise dataset is known to improve accuracy and specificity of the ROC analysis as well as improve predictability of the model [15, 20, 23]. In the registry, details that were not identified in variables such as G6PD, case locality, status of case locality, previous malaria infection, presence of gametocyte during admission, severity, and status of primaquine treatment were categorised as unknown. The lack of specifics could contribute to the moderate ROC values and hence a thorough data collection is required prior to data input in the malaria registry. Additional information not available in the current work such as co-morbidities, use of other medications, and type of previous malaria infection may also affect the positivity or negativity of the test results, and could be included to improve ROC values in the future. Generalisation of the current study should also be done with caution. Due to the differences in population demographics, and clinical and antimalarial-treatment management in Malaysia, compared to other regions, the results of the scores may differ.

## Conclusions

The current study was successful in developing a simple *P. vivax* recurrence prediction-tool. To the best of our knowledge, this is the first scoring system that is able to predict patients who are at a high risk of recurrence in Malaysia. The use of the scoring system in clinical settings would require minimal training. Furthermore, it is also ideal for use in field-trips in poorly developed areas. The incorporation of this clinical prediction-tool into the current 1-year follow-up protocol may further strengthen monitoring and future eradication of *P. vivax* infection in Malaysia.

## Abbreviations

ACT: Artemisinin-based combination therapies
BFMP: Blood film for malaria parasites
CP: Chloroquine
G6PD: Glucose-6-phosphate dehydrogenase
NMRR: National Medical Research Register
PQ: Primaquine
ROC: Receiver operating characteristic
WHO: World Health Organization
